# J. A. Estlander’s Clinical Contributions

**Published:** 1880-04

**Authors:** Herman Mynter


### TRANSLATIONS.

---

### J. A. ESTLANDER'S CLINICAL CONTRIBUTIONS,

### TO THE KNOWLEDGE OF OSTEO-SARCOMA OF THE SUPERIOR MAXILLARY BONE, AND OF ITS TREATMENT BY RESECTION OF THE BONE.

FROM THE SWEDISH BY HERMAN MYNTER, M. D.

FOR the surgeon called to treat a disease as serious as osteo-sarcoma of the superior maxillary, and still more for the patient, it is, above all, necessary to know, on the one hand, what are the chances of a definite recovery, or at the least of the prolongation of life, if one undertakes the dangerous operation of resection; and, on the other hand, how long the patient will be likely to live, if it be not undertaken. In order to answer these two questions, it is necessary that the surgeon know the average duration of the malady if left to run its course without the operation; as, also, the condition of the subjects, the number of radical cures on the one hand; on the other, the average time between the operation and the relapse, or the death which follows. These data, obtained in various ways, are, up to this time, too little determined; the surgeon seeing the patient only during a limited period of the disease, most often during his sojourn at the hospital. Doubtless he can learn the history of the patient and of the affection before the time of consultation; but too often he loses sight of the patient from the time he leaves the hospital after the operation, or without it. The comparison of a sufficient number of cases, well studied from the first attack until their termination, favorable or fatal, is necessary, in order to judge of the value of the operation. It is this work that the author has attempted, and of which he gives an account in his work.

The number of cases treated by him reaches twenty; not many, but he was, at least, able to follow them all to the end, and he has taken very complete notes of the progress of the disease from the commencement until the time when the patients presented themselves for treatment. Following we have the detailed history of these twenty cases, of which fifteen were men, and five only were women. The age varied from twenty-seven to sixty-nine years, and the number of patients below the average age of forty-eight years, is about the same as the patients above that age. The author demonstrates that with all, the disease presented, in its entire course, a remarkable uniformity, and he has given a particular account of the symptoms.

In four cases no operation could be made. One might add a fifth patient who was subject to an operation, but the tumor had penetrated the cranial cavity, and the patient would not probably have lived more than fifteen days. The duration of these cases, from the first attack until death, varied from five to nineteen and three-fourths months; the average is nearly a year. The cases operated had about the same duration. The progress of the disease has been generally a little more rapid with younger than with older subjects. The microscopic structure of the tumor did not appear to exercise a marked influence upon the rapidity of the disease, the *neoplasma*, with spindle-shaped cells, being no more mild than those with round cells. Besides, the two species co-exist in the same tumor, developing one after the other. A structure more areolar, and containing more connecting tissue, seems to indicate a more favorable condition.

As to the most essential part of the work, the prognosis of the resection, the author thinks one ought not to be too saving in operating. He rejects after his own experience, unfortunately in the commencement of his surgical career, partial resection for osteo-sarcoma of the superior maxillary, and he expresses the opinion that one rarely has occasion to regret the removal of too much, but too often that he has removed too little. In sixteen cases, two patients died shortly after the operation, their death not being necessarily attributed to it, (in one case meningitis, in the other simple pyæmia, supervened when the wound was almost closed). In one case death was due to an intervening malady; in a fourth, the result was still undecided.

Twelve cases remain from which to judge of the value of the operation. In two of these there has been a final cure; in two others a relapse, not *in loco* but in the glands of the neck, and the lives of the patients have been prolonged for a year. In the others, the relapse occurred *in loco*, and nothing was gained by the operation. With one of the subjects who recovered, the disease had not made much progress; but with the second, it had advanced considerably.

The study of all the cases which the author describes, leads to the conclusion, which seems quite strange at first, that in general the patients presented themselves to the surgeon just midway between the first stage of the disease and its termination; or in other terms, that the time, from the first appearance of the affection until the consultation, is about the same that remains to the patient to live; and this whether he be operated upon or judged unfit for an operation, (cases operated upon with success not counted).

Thus, when a subject has suffered from the disease six months before the presentation, or the operation, he would probably live six months; if he presented himself sooner—and for the reason that the disease makes more rapid progress, it would have the same rapid course, after the operation, and he would die sooner. The operation, if not followed by complete success, observes this law so far as the cases of the author permit him to judge. It seems only that the sufferings of the patient are less severe, and death less fearful in character with the operation than it would have been without it. But at what price do the sufferers gain this advantage? In the first place, it is necessary to consider the immediate danger of the operation. Even if one admits with the author, that these two cases of death ought not to be attributed to the operation, it is certain, and the author does not deny it, that the resection of the superior maxillary, is a serious operation, liable to produce death. There have been cases in which fatal hemorrhage has occurred by the introduction of blood into the air passages, and to obviate this peril it has been proposed that the previous operation of tracheotomy should be performed, a Trendelenburg's canula being introduced into the trachea. The author has never had recourse to this aid, believing it to be quite superfluous if one operates rapidly and regularly; but yet there are other dangers to be avoided. Briefly, there is some danger, but it is more than counterbalanced by the chances of a radical cure. The pain of the operation may be avoided, or greatly alleviated by anæsthetics, and after the operation, there is no suffering. For a time he is completely delivered from the distress previously experienced. The time demanded for the healing of the exterior wound, is short, and at the end of five weeks usually the patient is able to resume his habitual occupation. The difficulty in eating soon passes, but there is an unavoidable disfiguration of the face, consequent upon the removal of the bone.

To sum up, one can predict that a patient attacked with osteo-sarcoma of the superior maxillary, would have, ordinarily, a year to live after the commencement of the disease. During the first six months, his sufferings would not be very acute, so that generally he would not apply to a surgeon until this time had expired. Then, if he did not submit to an operation, he would still have six months more of life. The same thing occurs, if the operation should not be successful. When the disease has made so rapid progress that the patient is forced to an operation his life is proportionately shortened. If he submits to an operation, he exposes himself to a certain danger, but with a hope of cure equal to one chance in six. He has the chance to prolong his life twice as long, but the probability is four times greater that he will neither gain nor lose so far as time is concerned; but that life will be rendered more endurable during his few remaining months.

Osteo-sarcoma of the superior maxillary is after all as terrible a disease as other malignant diseases with which mankind is afflicted. There is little hope that its prognosis can be bettered by the progress of art; but it is possible, by the instruction of the people as to its nature and its danger, to induce them to apply more promptly to the surgeon, that an operation may be performed in the earlier stages of the disease, when the possibility of complete recovery is much stronger.—*Nordisk Medicinsk Archiv*, *Comptes-rendus.*

